# Hospital Economics

**Published:** 1916-12

**Authors:** 


### Hospital Economics.

No one will dispute the general statement that there is a demand for relatively cheap hospital care of patients. Private room accommodations at somewhere between $12 and $20 a week, in addition to professional fees and incidental losses, are obviously a heavy strain on incomes of from the same to twice these amounts. When half to all of the family income is expended on one member of the family, with only a slight proportionate saving in food which is counterbalanced by an incidental increase of general domestic upkeep, it needs no argument that we are dangerously near the breaking point at which the family is driven in to more or less honest pauperism, with a permanent abandonment of ideals of thrift and old-age saving, if such had been entertained. Unquestionably, the financial strain of the first baby has made dead beats of young men of originally honest intentions but who had not fully comprehended the extent of the financial responsibilities imposed by marriage. Yet almost all young couples of moderate income agree that, merely from the financial standpoint, the hospital is preferable to the home care of a labor case.

The community, as the responsible provider for the dependent class, finds that it must either pay between $1.25 and $2 a day for the hospital care of persons whose total income may not actually exceed these figures and rarely reaches so great a per capita income, or must itself be a dependent upon the voluntary gifts of the public.

In no spirit of criticism of hospital management and with no assumption of the knowledge of how the problem is going to be solved or whether it can be solved at all, we feel that as a general matter of economics, civic and professional, the gravity of the problem should be realized and that at least an earnest attempt should be made to solve it. Even the proper self-interest of the profession, gives it a peculiar duty in this regard. Every man who has been the recipient of medical charity in a hospital or who has set aside the temptation to receive such charity at great sacrifice, is to a degree trained to demand subsequent free medical attendance under any circumstances, as a right. The Buffalo Health Dept. has recently published a reply to a protest against the general right of the physician to receive pay for his services. The fact that it has even been considered necessary to present any argument on this point is a serious indication of the trend of popular opinion. Whenever any considerable number of persons, however excused by ignorance and however illogical, conceive that medical attendance is something due them from the community, it will need a revolution of professional economics in a way already demonstrated by European precedents, to be highly undesirable to the majority of the profession itself and probably tending ultimately to a degradation of the profession, even from the standpoint of efficiency.

There are various reasons why we feel hopeful of the satisfactory solution of the problem of hospital fees. The strictly professional attendance involved is eliminated since it is no more expensive for the person who pays his own way than for home attendance and since it is freely given to the poor in the wards. Even the slight increment of interne assistance is distributed over a large number of individuals and amounts merely to the expense of board and washing and a nominal honorarium. Nursing is similarly susceptible of distributive economy and is paid for, to the degree of about 50% in instruction. The hospital is established by voluntary donations and is free from taxation. Heat, food, washing and supplies, including medicines, are purchased in quantities or maintained on a scale sufficient to secure a much lower ultimate cost than in private homes. We do not overlook the fact that the standards of living and care which would obtain in the homes of the very poor are not up to those which must be maintained in every properly conducted hospital simply from humanitarian motives, not to mention the fact that in giving their service, the attending staff have a right to demand that they shall be properly supplemented by other factors, without undue regard for economy. We have known of cases in which, properly, a single drug used, cost more than the entire amount paid by the city—but we have known of analogous cases in which this form of expense was not even justified by the laudable desire to acquire clinical experience.

We repeat that this article is not written in a spirit of criticism but it is vitally important to study the problem carefully and to solve it as completely as possible.

